# Induction of Antibodies in Rhesus Macaques That Recognize a Fusion-Intermediate Conformation of HIV-1 gp41

**DOI:** 10.1371/journal.pone.0027824

**Published:** 2011-11-30

**Authors:** S. Moses Dennison, Laura L. Sutherland, Frederick H. Jaeger, Kara M. Anasti, Robert Parks, Shelley Stewart, Cindy Bowman, Shi-Mao Xia, Ruijun Zhang, Xiaoying Shen, Richard M. Scearce, Gilad Ofek, Yongping Yang, Peter D. Kwong, Sampa Santra, Hua-Xin Liao, Georgia Tomaras, Norman L. Letvin, Bing Chen, S. Munir Alam, Barton F. Haynes

**Affiliations:** 1 Human Vaccine Institute, Duke University School of Medicine, Durham, North Carolina, United States of America; 2 Department of Medicine, Beth Israel Deaconess Medical Center, Children's Hospital, Harvard Medical School, Boston, Massachusetts, United States of America; 3 Division of Molecular Medicine, Children's Hospital, Harvard Medical School, Boston, Massachusetts, United States of America; 4 Vaccine Research Center, National Institute of Allergy and Infectious Diseases, National Institutes of Health, Bethesda, Maryland, United States of America; University of Alabama, United States of America

## Abstract

A component to the problem of inducing broad neutralizing HIV-1 gp41 membrane proximal external region (MPER) antibodies is the need to focus the antibody response to the transiently exposed MPER pre-hairpin intermediate neutralization epitope. Here we describe a HIV-1 envelope (Env) gp140 oligomer prime followed by MPER peptide-liposomes boost strategy for eliciting serum antibody responses in rhesus macaques that bind to a gp41 fusion intermediate protein. This Env-liposome immunization strategy induced antibodies to the 2F5 neutralizing epitope ^664^DKW residues, and these antibodies preferentially bound to a gp41 fusion intermediate construct as well as to MPER scaffolds stabilized in the 2F5-bound conformation. However, no serum lipid binding activity was observed nor was serum neutralizing activity for HIV-1 pseudoviruses present. Nonetheless, the Env-liposome prime-boost immunization strategy induced antibodies that recognized a gp41 fusion intermediate protein and was successful in focusing the antibody response to the desired epitope.

## Introduction

The membrane proximal external region (MPER) of HIV-1 gp41 contains the epitopes of broadly neutralizing antibodies 2F5 and 4E10 [1], [2], [3], [4] that are important targets for HIV-1 vaccine design. The MPER of gp41 is a highly conserved region, rich in aromatic residues, and its role in HIV-1 fusion is evident from studies showing that mutation of tryptophan residues in the MPER inhibits cell fusion and viral infectivity [5], [6]. Passively administered neutralizing antibodies 2F5 and 4E10 can protect against vaginal SHIV transmission [7] indicating that if induced in high titers, such broadly neutralizing antibodies could be effective against HIV-1 infection. However, MPER-specific broadly neutralizing antibodies are rarely made in HIV-1 infection [8], [9] or following HIV-1 envelope protein (Env) vaccination [10], [11], [12], [13]. Structural constraints that include transient exposure of neutralizing epitopes [14], [15], as well as immunological tolerance mechanisms [16] are explanations for inability to routinely induce 2F5 or 4E10-like antibody responses. Thus, there are two barriers to induction of MPER broad neutralizing antibodies that must be overcome; the transient exposure and poor immunogenicity of subdominant MPER neutralizing epitopes, and tolerance control of the B cells capable of responding to the MPER neutralizing epitopes [16], [17].

The high affinity binding of 2F5 and 4E10 mAbs to the membrane-displayed MPER followed a two-step encounter-docking model that was distinct from the binding pattern observed in MPER antibody interaction with gp41 epitopes in the absence of lipids [18], [19]. These and other data suggest that MPER residues are orientated or presented differently on a lipid bi-layer compared to free peptides [15], [19], [20], [21]. The lack of binding of a non-neutralizing MPER mAb 13H11, the binding site of which overlaps that of 2F5, to MPER peptide-liposomes [18], further highlights the differences in configuration of the MPER residues in peptide-lipid complexes when compared to MPER peptide in solution. In contrast, gp41 peptides exist in multiple conformations - unstructured, α-helical, or β-turn [22], [23], [24] and MPER peptides alone as immunogens generally are ineffective for the induction of neutralizing antibodies (reviewed in [25]). MPER peptides in micelles or in liposomes with membrane anchor tags are likely to be less flexible and more likely to adopt a relatively ordered conformation [19], [20], [21], [26]. Ofek *et al*., recently showed that stable scaffold structures could induce antibody responses that target the 2F5-bound peptide conformation [27]. However, to date, no studies have demonstrated induction of gp41 fusion intermediate antibodies by Env or peptide immunogens.

In this study we report that a prime-boost regimen of oligomeric gp140 protein and MPER peptide-liposomes was required to induce MPER binding serum responses specific for the 2F5 core gp41 epitope ^664^DKW amino acids in both guinea pigs and rhesus macaques. The induced antibodies in Rhesus macaques bound the DKW sequence of the nominal 2F5 epitope, and to a protein construct designed to mimic a gp41 fusion intermediate conformation.

## Results

### Immunogenicity of gp140 oligomer protein and MPER peptide liposomes immunogens in guinea pigs

Broadly neutralizing gp41 MPER mAbs 2F5 and 4E10 either do not bind or bind poorly to many Env gp140 oligomers [14], [18]. We have previously reported that MPER epitopes are indeed exposed in JRFL gp140CF Env protein allowing binding of 2F5 and 4E10 mAbs [18]. We, therefore, first immunized guinea pigs to determine if either JRFL gp140CF or MPER peptide-liposome complexes that both bind 2F5 and 4E10 mAbs could induce antibody responses to the MPER neutralizing epitope ^664^DKW.

**Figure 1:**
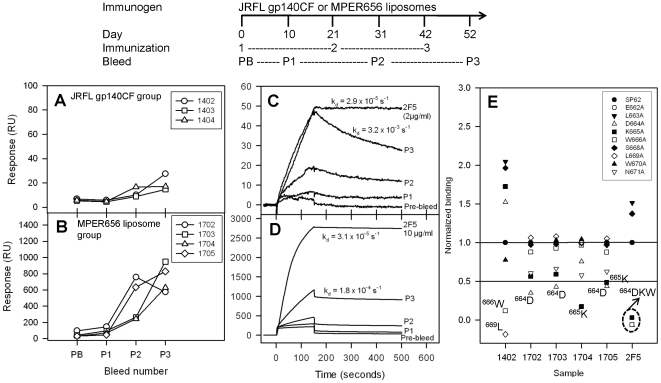
Comparison of gp41 MPER specific antibody responses induced in guinea pigs by JRFL gp140CF and MPER peptide-liposomes. The immunization scheme of the study is shown in the top panel. **A–B:** The 2F5 epitope peptide (SP62 peptide) specific responses in guinea pigs sera (at 1∶50 dilution) determined by SPR are shown after vaccination with JRFL gp140CF (A) and MPER peptide-liposome (B). PB, pre-bleed; P1–P3, post-immune bleeds 1–3. **C–D:** SPR sensogram displaying the comparison of 2F5 epitope peptide specific binding of guinea pig 1402 (C) and 1703 (D) sera at different time points with 2F5 mAb is shown as representative data. The estimated dissociation rates (k_d_) from the sensogram are indicated for 2F5 mAb and Post-bleed 3 (P3). **E:** Epitope mapping of post-immune bleed 3 of guinea pigs 1402 (immunized with JRFL gp140CF alone) and 1702–1705 (immunized with MPER656 peptide-liposomes alone) is shown in comparison to 2F5 mAb. The normalized binding shown is the ratio between binding responses of sera to the alanine scanning mutant peptides and WT 2F5 epitope peptide SP62. Alanine substitution of residues that resulted in ≥50% reduction in binding are indicated adjacent to the symbols. Data is representative of at least two measurements on adjacent spots on the same sensor chip.

Immunization of JRFL gp140CF oligomer protein alone (3×) induced low levels of weak avidity MPER specific responses as indicated by the 100-fold faster dissociation rate (k_d_) when compared to that of 2F5 mAb (Figure 1A&C). On the other hand, MPER peptide-liposome complexes immunized alone in guinea pigs induced relatively higher avidity antibodies as judged by relatively slower dissociation rates (∼6-fold faster in comparison to that of 2F5 mAb) (Figure 1B&D). Thus, compared to Env JRFL gp140CF protein, MPER peptide liposomes induced higher avidity 2F5 peptide-specific responses.

Next we determined the fine specificity of MPER specific responses induced by these two immunogens alone. Only guinea pig 1402 when immunized with JRFL gp140CF gave sufficiently high MPER responses. In serum from animal 1402, JRFL gp140CF immunization induced responses targeting ^666^W residue and an additional residue (^669^L) outside the 2F5 core (Figure 1E) that is a critical residue for a non-neutralizing MPER mAb [28]. The MPER peptide-liposome immunizations induced responses targeting residues ^664^D in two animals (1702 and 1703), ^665^K in one animal (1704) and both ^664^D and ^665^K in one animal (1705, Figure 1E). Thus, neither JRFL gp140CF nor MPER peptide-liposomes elicited responses with specificity focused entirely on the 2F5 core tripeptide ^664^DKW residues that are critically involved in mAb 2F5 neutralization.

**Figure 2:**
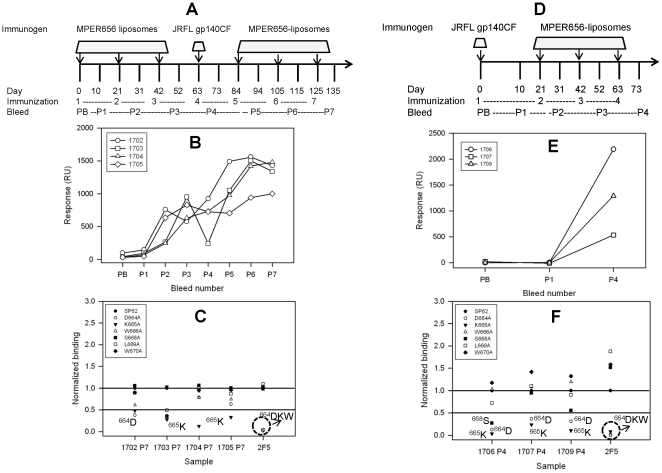
MPER specific immunogenicity and fine specificity of different prime-boost regimens in guinea pigs. **A–C:** Immunization scheme (A), 2F5 epitope peptide (SP62 peptide) specific binding serum responses (B) and fine specificity of post immune bleed 7 (C) are shown for MPER656 liposome prime-JRFL gp140CF boost regimen are shown. PB, pre-bleed; P1–P7, post-immune bleeds 1–7. **D–F:** The JRFL gp140CF prime-MPER656 liposome boost regimen scheme (D), 2F5 epitope peptide specific binding serum responses € and the fine specificity of post immune bleed 4 (F) are shown. Data is representative of at least two measurements on adjacent spots on the same sensor chip.

Since we observed weak 2F5 epitope specific responses with JRFL gp140CF alone and only partial focusing on the 2F5 core epitope with MPER peptide liposomes, we next asked whether a heterologous prime-boost regimen using both immunogens would induce antibody responses with specificity restricted to the core tripeptide ^664^DKW residues. To determine the optimal prime-boost regimen, we used two different strategies. First, we tested a MPER peptide-liposome prime-JRFL gp140CF boost regimen as shown in Figure 2A (same animals in Figure 1B were boosted). In the second strategy, we used a single JRFL gp140CF prime followed by MPER liposome boost (3×) as shown in Figure 2D. The MPER peptide-liposome prime-JRFL gp140CF boost regimen resulted in immune responses (Figure 2B) that targeted either ^664^D or ^665^K (Figure 2C) and were not different from the results obtained with MPER peptide-liposome alone immunizations. In contrast, as shown in Figures 2D–F, a single prime with JRFL gp140CF followed by MPER peptide-liposomes elicited 2F5 peptide specific responses (Figure 2E) that targeted two of the tripeptide core residues, ^664^D and ^665^K, in all three animals (Figure 2F). Thus, the strategy of priming once with JRFL gp140CF and boosting (3×) with MPER peptide-liposomes induced antibody specificity that was closer to that of the neutralizing antibody 2F5. In a separate study, the induction of 2F5-like specificity was reproducible and in one animal two rounds of prime-boost immunizations progressively focused the response to the 2F5 core tripeptide ^664^DKW residues (Figure S9).

### Non-human Primate (NHP) serum antibody responses to JRFL gp140CF and MPER peptide-liposomes

**Figure 3:**
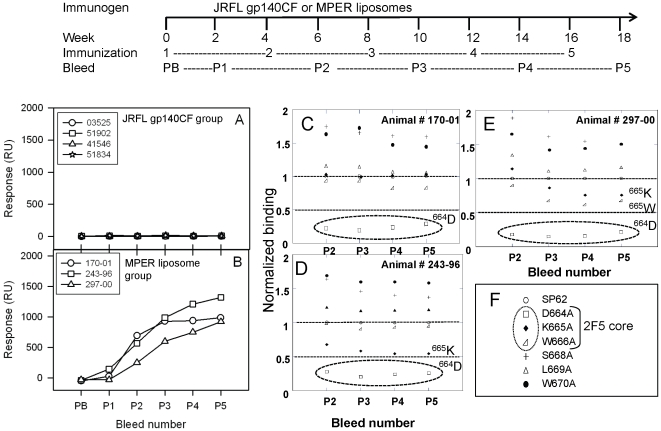
Comparison of MPER specific immunogenicity of different immunogens in rhesus macaques. The immunization scheme is shown at the top. **A–B:** MPER specific binding responses of sera from immunized rhesus macaques at different time points are shown for (A) JRFL gp140CF (ASO1_B_ adjuvant) and (B) MPER peptide liposome immunogens (in emulsigen+oCpG). **C–E:** The fine specificities of MPER specific responses elicited by MPER liposomes in rhesus macaques are shown for post immunizations 2–5 sera. **F:** The alanine substituted peptides used in epitope mapping of MPER responses. The dotted ellipses in C, D and E highlight the predominance of ^664^D specific MPER responses. Data is representative of at least two measurements on adjacent spots on the same sensor chip.

To determine if the strategy of gp140 prime/MPER peptide-liposome complex boost for induction of anti-^664^DKW antibodies would translate to non-human primates, we next examined the immunogenicity of JRFL gp140CF alone and MPER peptide-liposomes alone in rhesus macaques. Immunization of JRFL gp140CF oligomer alone induced weak (<1∶100 titer) MPER-specific responses in rhesus macaques (Figure 3A) although end-point antibody titers against the JRFL gp140CF Env protein were ≥1/10,000 (Figure S1). In contrast, MPER peptide-liposome complexes alone did induce MPER-specific antibodies (Figure 3B), but the specificity of the MPER liposome-induced responses was restricted to residue ^664^D, with weak and inconsistent involvement of ^665^K of the 2F5 core tripeptide ^664^DKW (Figure 3C–E). Thus, antibody responses with the desired amino acid targeting (^664^DKW) were not achieved in rhesus macaques immunized with MPER peptide-liposomes or JRFL gp140CF oligomer alone.

### Rhesus macaque antibody responses to heterologous prime-boost immunization

**Figure 4:**
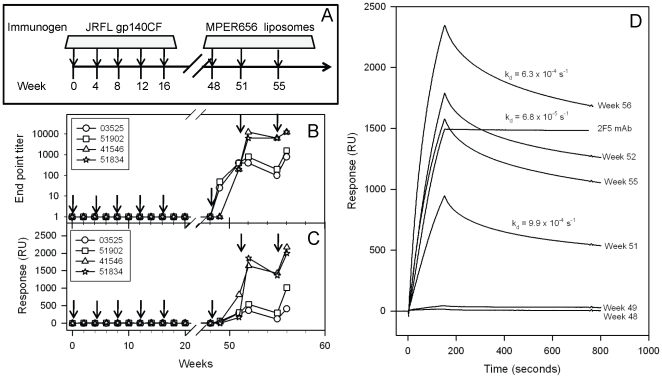
MPER specific binding responses of Rhesus macaques sera in gp140 prime-MPER liposome boost regimen. **A:** The immunization scheme involving JRFL gp140CF prime (ASO1_B_ adjuvant) and MPER656/MPL-A/R848 (emulsigen+oCpG) liposome boost regimen is shown. The arrows indicate the weeks at which immunization was done. **B:** ELISA end point titer of the 2F5 epitope peptide specific responses in rhesus macaques sera at different time points. **C:** The 2F5 epitope peptide specific binding of monkeys sera determined by SPR is shown post vaccination at different time points. **D:** Representative data of SPR sensogram of rhesus macaque #41546 sera at different time points binding to 2F5 epitope peptide as compared to 2F5 mAb (10 µg/ml) is shown. The estimated dissociation rates (k_d_) are indicated for samples at week 51 and 56 as well as for the control 2F5 mAb. Data is representative of at least two measurements on adjacent spots on the same sensor chip.

Since we observed that heterologous prime-boost immunizations in guinea pigs were more effective in inducing ^664^DKW -specific antibody responses, we next tested whether the JRFL gp140CF prime-MPER peptide-liposome boost regimen would also focus the induced antibody responses on the gp41 ^664^DKW neutralizing epitope in rhesus macaques. The Env prime, MPER-liposome boost immunization regimen used in rhesus macaques is shown in Figure 4A (same animals in 3A were boosted). The MPER specific responses in sera from four immunized animals at different points are shown in Figures 4B–C. MPER specific responses began to appear in sera collected 3 weeks after first MPER peptide-liposome complex boost (week 51). Each subsequent boosting enhanced the level of 2F5 peptide binding responses, with two animals having about 3-fold higher binding responses than the remaining two (Figure 4B–C). The responses declined during the interval between the 2^nd^ and 3^rd^ boost but then increased again following the 3^rd^ MPER peptide-liposome complex boost (Figure 4B–C). Serum antibody binding dissociation rates, a measure of binding avidity, was stable over time (Figure 4D).

**Figure 5:**
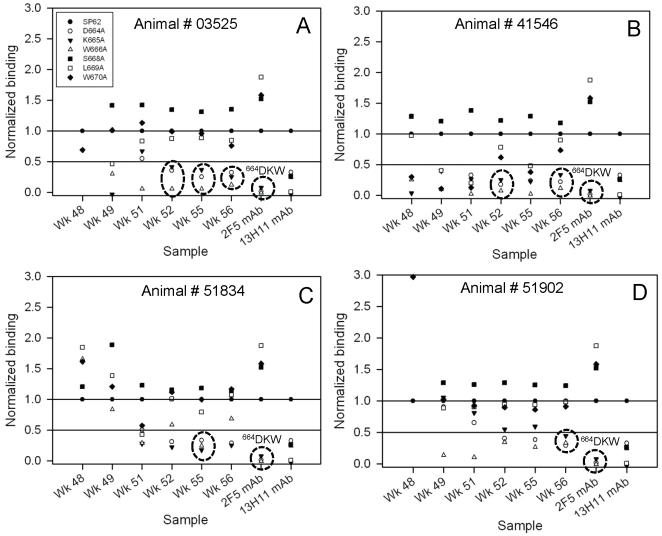
Mapping of critical residues involved in MPER specific Rhesus macaques serum responses. Normalized binding responses of immune sera from rhesus macaques primed with JRFL gp140CF and boosted with MPER656 liposomes. Panels A–D are for animals 03525, 41546, 51384 and 51902. The dotted circle highlights the mapping of MPER specific responses to D^664^KW residues as did 2F5 mAb. Data is representative of at least two measurements on adjacent spots on the same sensor chip.

Fine specificity mapping of induced antibody demonstrated that the MPER-specific responses elicited after the first boost of MPER liposomes were targeted to one or two core ^664^DKW residues and as well targeted additional MPER residues (L669, W670), outside the 2F5 core but within the MPER hinge region (Figure 5). However, following the second and third MPER peptide-liposome complex boosts, rhesus monkey serum antibody responses to ^664^DKW residues were consistently induced (Figures 5A–D). In 3 out of 4 animals (03525, 41546 and 51902), sera collected one week after the third boost (week 56) showed MPER specific responses in which all three ^664^DKW residues were recognized by serum antibodies (Figures 5A,B and D). In the fourth animal (51834), the specificity for all three DKW residues was also observed in week 55 bleed but with weaker and transient involvement with W^666^ in week 56 (Figure 5C). Thus, these results indicated that using a heterologous prime-boost regimen of JRFL gp140CF/MPER peptide-liposome complexes, MPER-specific antibody responses in rhesus macaques could be focused to MPER ^664^DKW amino acids.

### Induction of antibodies to gp41 fusion-intermediate protein by Env gp140 prime, MPER liposome boost regimen

**Figure 6:**
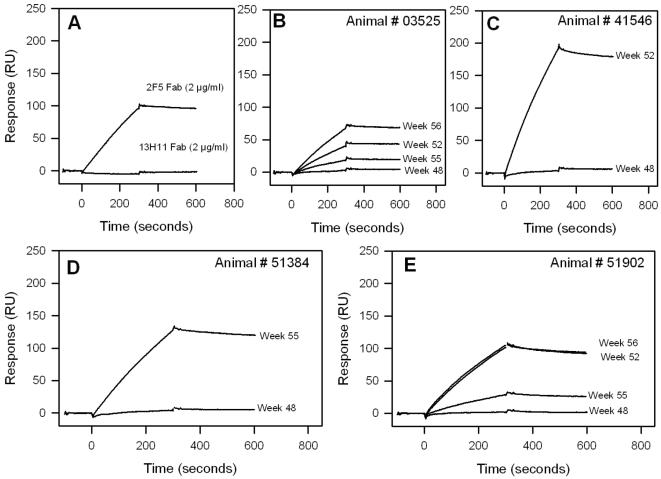
The MPER specific serum responses in rhesus macaques recognize the mimic of fusion-intermediate gp41 structure. **A:** Comparison of binding of 2F5 and 13H11 Fabs to GCN4-gp41-inter, a mimic of pre-hairpin intermediate structure of gp41. **B–E:** SPR sensogram of rhesus macaques sera (1∶50 diluted) binding to GCN4-gp41-inter. Each panel in B thru E compares the binding of sera obtained prior to MPER656 liposomes boost (week 48) and post-MPER656 liposomes boost (weeks 52–56). Data is representative of two measurements.

**Figure 7:**
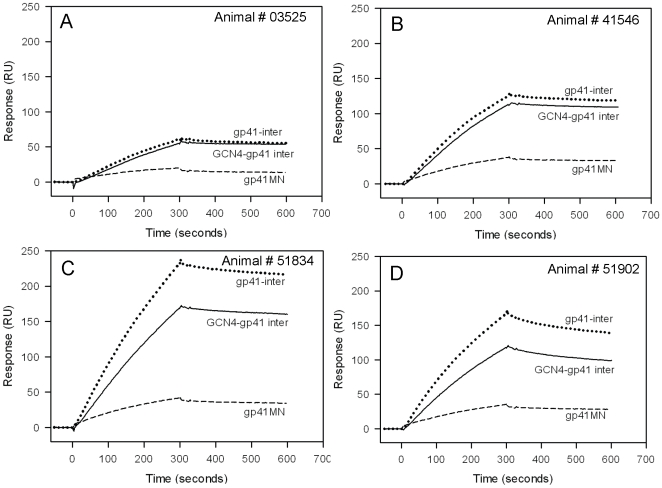
Comparison of JRFL gp140CF primed and MPER656 liposomes boosted rhesus macaque serum IgG binding to different conformational states of gp41. **A–D:** SPR sensogram of rhesus macaques serum IgG (100 µg/ml) binding to GCN4-gp41 inter (solid lines), gp41-inter (dotted lines) and recombinant gp41 (broken lines). The IgGs purified from Week 56 (for animals 03525 and 51902), Week 52 (animal 41546) and Week 52 (animal 51834) sera were used. Data is representative of two measurements.

While induction of pre-hairpin intermediate antibodies is difficult, induction of post-fusion six-helix bundle antibodies is common [17], [29], [30], [31]. The neutralizing MPER antibodies 2F5 and 4E10 target the fully extended fusion intermediate state of HIV-1 gp41 [14], [32] whereas non-neutralizing MPER antibody 13H11 recognizes a helical MPER structure that is consistent with HIV-1 gp41 in a post-fusion six-helix bundle conformation [28]. To fully understand the gp41 conformation targeted by the induced antibodies, we tested three different recombinant gp41 proteins; the gp41MN protein, and two gp41-inter constructs- gp41-inter and GCN4 gp41-inter [14], [32]. While both trimeric gp41 inter proteins present the MPER in its putative fusion intermediate conformation, gp41-inter construct has an additional six-helix bundle attached at its N-terminus end [14], [32]. Thus antibodies that exclusively recognize the fusion intermediate conformation of gp41 will bind to both the gp41 inter proteins, while those that do not target the fusion intermediate will bind to gp41 and/or gp41-inter but not GCN4-inter [32]. JRFL gp140CF immunized macaques sera (week 14, Figure S2), which gave no MPER peptide binding, bound to both gp41 MN and gp41-inter but not to GCN4 gp41-inter. The gp41 specific antibodies induced by JRFL gp140CF, therefore, are unable to recognize the fusion intermediate conformation of gp41. Similarly, the week 48 sera (following priming with JRFL gp140CF protein), which showed no detectable MPER specific responses by SPR, also gave no binding to GCN4-gp41 inter (Figure 6B–E). Remarkably the post-MPER656 liposome boost sera (Weeks 52–56) that gave DKW-specific responses bound strongly to GCN4-gp41 inter (Figure 6B–E). Since GCN4-gp41 inter is recognized by 2F5 and not by 13H11 (Figure 6A), the GCN4-gp41 inter binding of MPER specific responses in these rhesus macaques sera indicated that the induced DKW-specific antibodies were capable of recognizing the fusion-intermediate state of gp41. Furthermore, as shown in Figure 7, the purified IgG binding responses gave higher binding to both gp41-inter and GCN4-inter while the binding to gp41 MN was much lower. These results indicated that MPER peptide-liposomes boost can focus the antibody responses to the 2F5 core DKW residues and induce antibodies in rhesus macaques that preferentially bound the MPER conformation in the fusion-intermediate protein. Thus, the JRFL gp140CF/MPER peptide-liposome complex regimen has solved one component of the problem for induction of neutralizing anti-gp41 MPER antibodies, i.e., induction of antibodies with specificity for a fusion-intermediate conformation of gp41 MPER.

### JRFL gp140CF prime, MPER liposome boost regimen induced antibodies bind to 2F5-epitope scaffold proteins

**Figure 8:**
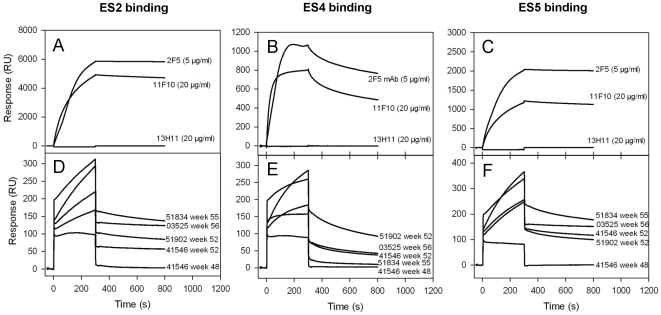
The JRFL gp140CF primed and MPER656 liposomes boosted rhesus macaque serum IgG bind scaffold proteins containing engrafted MPER in the 2F5 bound conformation. A–C: SPR sensogram of 2F5, 11F10 and 13H11 mAbs at the indicated concentrations binding to ES2 (A), ES4 (B), and ES5 (C) scaffolds respectively are shown. **D–F:** The post MPER656 liposome immune serum IgG of rhesus macaques (150 µg/ml) binding to ES2 (D), E€(E), ES5 (F) are shown. For the sake of clarity, the scaffold binding responses of pre-MPER656 liposome serum IgG of only one animal (# 41546) is shown. The responses from other animal serum IgG were similar. Data is representative of at least two measurements.

We next asked whether the JRFL gp140CF prime:MPER656 liposome boost regimen induced antibodies recognize the 2F5 epitope in the 2F5-bound conformational state. Ofek et al recently demonstrated that computationally designed 2F5-epitope scaffolds that had the epitope in a 2F5-bound conformation possessed nanomolar affinity for 2F5 mAb with varying degrees of binding entropy [27]. Crystal structure analysis of one of the scaffold (ES2) which had the highest affinity towards 2F5 and the most entropically favorable interaction with 2F5 showed that the epitope conformation mimicked that of the 2F5-bound conformation [27]. Thus, we examined the binding of pre- (week 48) and post-MPER656 liposome (weeks 52–56) immune serum IgG of rhesus macaques to 2F5-epitope scaffolds ES2, ES4 and ES5. As previously reported [27], 2F5 mAb and 11F10, a mouse monoclonal antibody elicited in a ES5–ES1 prime∶boost immunization regimen bound strongly to the ES proteins (8A–C). The non-neutralizing 13H11, which recognizes the 2F5 epitope core (^664^DKW) and L669 [15] in a helical conformation [28] did not bind the 2F5-epitope scaffolds (Figure 8A–C). Interestingly, the post-MPER656 liposome immune serum IgG of rhesus macaques bound to 2F5-epitope scaffolds ES2, ES4 and ES5 (Figure 8D–F). As also observed with GCN4-gp41-inter binding, the post JRFL gp140 induced antibodies (week 48) did not bind to any of the scaffolds (Figure 8D–F).

To determine if the ES2 scaffold shares antigenicity with the gp41 inter proteins, we determined the ability of the 11F10 mAb to bind to gp41-inter proteins. We found that 11F10 bound to gp41 inter proteins (K_d_ = 7.9 and 7.8 nM to GCN4-gp41 inter and 92Ug gp41-inter respectively), but not to the gp41 MN protein (Figure S3). 11F10 mAb, therefore, selectively binds to the putative fusion-intermediate conformation of gp41. However, unlike 2F5 mAb 11F10 did not bind anionic lipids (Figure S4) nor neutralized HIV-1 [33]. Thus, these data indicate that the ^664^DKW specific antibodies elicited in rhesus macaques can not only recognize fusion-intermediate protein but can also bind to 2F5-bound conformation of gp41 MPER.

### Neutralization capacity of rhesus monkey serum antibodies that bind to gp41 fusion-intermediate protein

Having elicited ^664^DKW-specific antibodies that recognize the gp41 fusion-intermediate conformation and 2F5-bound conformation in rhesus macaques, we asked whether these responses would neutralize HIV-1. In TZM/bl pseudovirus assays (Table-1) no significant neutralization was detected in rhesus macaque sera taken at week 56 when the ^664^DKW core antibody responses were observed.

**Table 1:** Neutralization data of sera from JRFL gp140CF-MPER656 liposomes immunized rhesus macaques.

Animal #	ID50 for BG1168.1	ID50 for SF162.LS
03525	<20	<20
41546	<20	<20
51902	<20	<20
51834	<20	22
Positive control		
2F5 mAb	2.4 µg/ml	3.6 µg/ml

Sera from immunized guinea pigs did not neutralize (data not shown).

The lipid reactivity of 2F5 and 4E10 through the hydrophobic residues in the CDR H3 loop is critical for HIV-1 neutralization but not for antigen binding [29]. Thus, we asked whether the GCN4-gp41 inter binding ^664^DKW-specific antibodies elicited in rhesus macaques had any lipid reactivity. Purified IgG from rhesus macaque sera at weeks 0, 48, 52, and 55 was tested for reactivity to liposomes containing cardiolipin, or phosphatidylserine (PS) that do bind to mAb 2F5 [34]. There was no specific binding of rhesus macaque IgG purified from either the pre- or the post-vaccination sera to either cardiolipin (Figure S5) or PS (not shown). Taken together these results suggested that the induced DKW-specific rhesus macaque antibodies lacked the ability to bind lipids, and were therefore, unable to neutralize HIV-1.

## Discussion

In this study, we report that the membrane bound form of gp41 MPER peptides can present epitopes in a conformation that induce antibodies that not only target the core ^664^DKW epitope of the neutralizing antibody 2F5, but also recognize a fusion-intermediate construct of HIV-1 gp41 MPER as well as the 2F5 bound MPER conformation. The induction of antibodies with specificity for 2F5 tripeptide core residues was acquired only after priming with an oligomeric gp140, followed by multiple boosts with MPER liposomes, implying that successive boosting was critical for the expansion and maturation of B cells with receptors that target the core ^664^DKW MPER epitope. Although the induced responses were not neutralizing, the success of this immunization strategy to induce a putative fusion intermediate/2F5 bound conformation recognizing antibodies provides a solution to one barrier to inducing gp41 neutralizing antibodies.

One explanation for the failure of earlier vaccines is that the immunogens used were not configured in the conformation needed to elicit a gp41 fusion-intermediate antibody response. Our current regimen overcomes this problem. However, during HIV-1-induced CD4 T cell fusion, the MPER is only exposed transiently [14]. In order to capture such a transient epitope we have hypothesized that elicited antibodies should be capable of concentrating on virion surfaces by weak interactions with viral membrane lipids [29]. We have also hypothesized that such antibodies might not be easily made since lipid or protein autoreactivity of MPER specific antibodies may evoke tolerance controls [34]. Indeed, this has been shown to be the case in 2F5 VH knock-in mice [16], and in 2F5 VH+VL knock-in mice (Verkoczy L and Haynes B, unpublished).

Several earlier studies have used specialized structures to present the MPER sequences in the immunogens. Arnold *et al* had used recombinant human rhinoviruses displaying ELDKWA epitope flanked by residues promoting β- turn [35], Zhang et al expressed chimeric virus like particles presenting a bovine papillomavirus L1-HIV-1 gp41 fusion protein [36] and Liang et al had grafted ELDKWA into variable loops in HIV-1 gp120 [37]. These and other studies have reported induction of antibodies that target the 2F5-nominal epitope but with minimal or no neutralization [24], [35], [36], [37], [38], [39], [40]. Although in these studies, induced antibody responses were targeted to the ELDKWA sequence, fine specificity mapping data are not available for determination if the antibody responses were restricted to the core tripeptide ^664^DKW. Similarly, in studies with immunogens with 4E10 epitope, the lack of fine specificity data does not allow assessment of the epitopes induced by the immunogens [13], [41]. More recently, however, MPER directed neutralizing antibodies were reported to be elicited using immunogens consisting of gp41 six helix bundle and an exposed MPER tail with double mutation [42] and a chimeric construct in which the HIV gp120 was replaced with HA1 subunit of influenza virus [43]. That weak MPER neutralizing activity can sporadically be induced and detected is not surprising, since a subset of chronically HIV-1 infected subjects have been reported to make neutralizing antibodies that bind within the MPER [9], [44], [45]. However, high and sustained levels of MPER antibodies are not induced in the setting of vaccination and this may relate to immunoregulatory control of poly reactive MPER antibodies with more potent neutralizing activity such as 2F5 and 4E10 [16], [46].

Guenaga et al. [33] and Ofek and colleagues [27] recently demonstrated that a heterologous scaffold prime-boosting strategy induced antibody responses to the engrafted 2F5 epitopes, and that these antibodies bound to the MPER peptide that existed in the 2F5 bound conformation. These latter studies did not determine reactivity with gp41 fusion-intermediate forms, nor did these scaffold-induced antibodies neutralize HIV-1. However, like the MPER liposomes induced antibodies described in this study, one antibody, 11F10, isolated from the above 2F5 scaffold murine immunization study [27] also bound preferentially to the gp41-inter conformation and not to the recombinant gp41 MN (Figure S3). In this study we have described the induction of antibodies that preferentially bind to a gp41 fusion- intermediate protein and showed that such antibodies are also capable of binding to 2F5-epitope that is pre-configured in the 2F5 antibody bound state. Thus both immunization strategies, that of Ofek et al. (30) and the present study, have elicited antibodies that recognize the putative fusion-intermediate gp41 conformation. This is underscored by the fact the epitope scaffold induced antibody 11F10, which recognizes the 2F5 bound conformation of gp41, can also recognize the putative gp41 fusion-intermediate conformation as presented in the constrained gp41-inter protein.

It is notable in the studies of Ofek et al [27] and Guenaga and colleagues [33] that 2F5 scaffolds that were more flexible were better immunogens than those that were rigid. Thus immunogens that allow antigen recognition with induced fit may allow better adaptability to the available naïve B cell repertoire. Our studies using a heterologous prime boost and with MPER presented on liposomes provide an alternative strategy to inducing antibodies with specificity for the 2F5 core epitope, and in all likelihood allowed induced fit binding of 2F5 core epitope-specific antibodies. Importantly, the antibodies induced by our heterologous prime and boost preferentially bound gp41 fusion- intermediate constructs versus the recombinant gp41 MN. Thus, we have demonstrated that in addition to 2F5 stabilized structures engrafted on acceptor proteins, MPER liposomes when used as a boost can induce gp41-inter protein specific antibodies.

It is tempting to speculate that the MPER presentation in JRFL gp140CF is capable of priming for ^664^DKW responses but this response remains subdominant in the presence of other immunodominant gp140 epitopes. We have shown that JRFL gp140CF primarily induces antibody responses that target the recombinant gp41 MN and not the GCN4-gp41 inter protein (Figure S2). We postulate that B cells to the MPER ^664^DKW were primed by JRFL gp140 and then were expanded when boosted with MPER peptide-liposome complexes lacking other immunodominant epitopes. Although the 2F5 epitope portion of MPER sequence of the priming Env gp140 does not differ (Figure S6) from that used in the MPER liposomes (boost immunogen), the gp140 MPER likely presents a different structure than those on the MPER peptide liposomes. This is evident in the differences in binding affinities of 2F5 or 4E10 to JRFL gp140CF and MPER liposomes [18], [19] and in the nature of the induced antibodies (Figure 7 and S2). The heterologous prime/boost strategy with the two MPER structures have thus likely led to the focusing of the antibody responses to the 2F5 core residues and thus resulted in the induction of gp41 inter specific antibodies. Additionally, incorporation of TLR 4 and 7/8 agonists in the MPER peptide-liposome complexes also may have played a role in enhancing immune responses in the boosting stage. In this regard, we have previously reported the partial control of non-neutralizing gp41 MPER antibodies by tolerance controls [17], and others have demonstrated that combinations of TLR agonists including oCpGs and R848 have broken peripheral tolerance and induced autoreactive antibodies [47], [48], [49], [50]. Thus, strong adjuvants and repetitive boosting can overcome some of the constraints for immunogenicity of the 2F5 epitope of the MPER, but strategies for the induction of the polyreactive antibodies needed for neutralizing activity have to be developed. In this regard, Matyas et al have shown that liposomes containing the MPER and lipid A induced polyreactive IgM but not IgG antibodies that simultaneously bind gp41 and phosphatidylinositol-4-phosphate and neutralized HIV-1 in a peripheral blood mononuclear cells neutralization assay [51].

That the prime boost regimen reported here and constrained scaffolds can induce 2F5 core ^664^DKW epitope raises the question of the utility of immunization with a gp41-inter protein constructs. The prototype gp41 intermediate construct also contained the post-fusion 6 helix bundle [14], [32], and immune serum from guinea pigs immunized with this construct did not focus antibodies on ^664^DKW residues (Alam, M, Chen, B. et al. unpublished data). Studies are underway with a GCN4 stabilized gp41 pre-hairpin intermediate construct that does not contain the post-fusion six-helix bundle.

Finally, these results indicate that mounting of responses to 2F5 core epitope as it exists in the fusion intermediate conformation of gp41-inter alone is insufficient for HIV-1 neutralization. We have previously shown that mutation in the 2F5 CDR H3 that abrogate 2F5 binding to lipids but maintain binding to the pre-hairpin gp41 intermediate conformation are unable to neutralize HIV-1 (2). Thus, it is likely that the induced DKW-specific antibodies described in this study also lack a hydrophobic CDR H3 loop that renders them incapable of reacting with viral membrane lipids and are thus unable to neutralize. This is consistent with the results that 2F5 CDR H3 mutations greatly reduced the binding of recombinant 2F5 to MPER peptide liposomes (Figure S8-A). 11F10 mAb, which lacks a hydrophobic CDRH3 [27], also gave weak binding to MPER peptide liposomes (Figure S8-B). We had proposed that the lack of lipid binding of 2F5 CDR H3 mutants would prevent them from pre-concentrating on the viral membrane and such antibodies would fail to capture the transient fusion intermediate state of gp41 or to neutralize. Thus, while IgGs from immunized rhesus macaques showed some binding to MPER peptide-liposomes (Figure S8 C–F), the lack of lipid binding would prevent the rhesus IgGs to maintain a kinetic head start required for HIV-1 neutralization.

## Materials and Methods

### Ethics Statement

The guinea pig studies were carried out following a protocol (A334-09-11) approved by the Animal Use and Care Committee of the Duke University IACUC, which ensures that all animals in experimental research are used appropriately and are treated with the highest standards of humane care. Colony bred Indian-origin rhesus monkeys used in the immunization studies were housed and maintained in an Association for Assessment and Accreditation of Laboratory Animal Care-accredited institution in accordance with the principles of the National Institute of Health. All studies were carried out in strict accordance with the recommendations in the Guide for the Care and Use of Laboratory Animals of the National Institutes of Health in BIOQUAL (Rockville, MD). BIOQUAL is fully accredited by AAALAC and through OLAW, Assurance Number A-3086. The animal protocol used in this study was approved by the BIOQUAL IACUC (#08-3449-71). All physical procedures associated with this work were done under anesthesia to minimize pain and distress in accordance with the recommendations of the Weatherall report, “The use of non-human primates in research”. Teklad 5038 Primate Diet was provided once daily by animal size and weight. The diet was supplemented with fresh fruit and vegetables. Fresh water was given ad libitum.

### Proteins, Peptides, mAbs, Phospholipids and Adjuvants

Recombinant JRFL gp140CF (C = gp120−gp41 cleavage site deleted; F = fusion domain-deleted, Figure S6) was produced and purified using methods described earlier [52]. MPER peptides containing the 2F5 epitope (QQEKNEQELLELDKWASLWN) or the epitopes of both 2F5 and 4E10 mAbs (NEQELLELDKWASLWNWFNITNWLWYIK) were synthesized with a C-terminal hydrophobic membrane anchor tag (YKRWIILGLNKIVRMYS) and purified by reverse phase HPLC. The purity of the custom made (CPC Scientific) MPER peptides were assessed by HPLC to be greater than 95% and confirmed by mass spectrometric analysis. The gp41 inter proteins (92UG gp41-inter and GCN4-gp41 inter, Figure S7) having gp41 sequence of clade A HIV-1 isolate 92UG037.8 were produced as described earlier [32]. Chloroform stocks of phospholipids 1-palmitoyl-2-oleoyl-sn-glycero-3-phosphocholine (POPC), 1-palmitoyl-2-oleoyl-sn-glycero-3-phosphoethanolamine (POPE), 1-palmitoyl-2-oleoyl-sn-glycero-3-phosphoserine (POPS), 1,2-dimyristoyl-sn-glycero-3-phosphate (DMPA), cholesterol (CH), monophosphoryl Lipid A (MPL-A) and bovine heart cardiolipin were obtained from Avanti Polar Lipids and were used without further purification. R848 was purchased from Axxora LLC. The custom made oCpG with 5′ TCGTCGTTGTCGTTTTGTCGTT 3′ (used in guinea pigs immunization) and 5′-(P = S)TCGTCGTTTTTCGGTCGTTTT-3′ (used in rhesus macaques immunization) sequences was obtained from The Midland Certified Reagent Co. Emulsigen was purchased from MVP Laboratories. 2F5, 4E10 mAbs were obtained from Polymun and Synagis was obtained from Medimmune. 13H11 mAb and Fabs of 2F5 and 13H11 were produced as described earlier [28], [52]. Recombinant gp41 MN was obtained from Immunodiagnostics. The recombinant 2F5 mAb and the mutants were produced in 293T cells as described earlier [29].

The epitope scaffold proteins ES2, ES4 and ES5 and the 11F10 mAb were made as described earlier [27]. For antibody preparation, 250 µg of light chain plasmid DNA and 250 µg of heavy chain plasmid DNA, or for ES2 and ES4 scaffolds, 500 µg plasmid DNA, were mixed with 1 ml of 293fectin (Invitrogen, Carlsbad, CA) for 20 minutes before the DNA-293fectin complex was added into 850 ml of FreeStyle 293F cells (1.4×10^6^ cells/ml) in a 2-L shaking flask. The transfected cells were returned to suspension incubation for 2 days, and then, the culture was fed with 50 ml of protein expression enriched medium CellBoost-5 (HyClone, Logan, UT) and protein expression enhancer Sodium Butyrate at final concentration of 2 mM (SIGMA, St. Louis, MO). After 6 days post transfection, supernatants were harvested by centrifugation, filtered through 0.22 µm filter. The 11F10 antibody was purified by running the supernatant over a protein A column (Pierce Protein A Plus Agarose, Thermo, Rockford, IL), followed by elution at low pH. Purified antibody was dialyzed against PBS, analyzed SDS-PAGE and stored at −80°C. The ES2 and ES4 scaffolds were purified by running the supernatant over a 2F5 antibody affinity column, followed by elution at low pH. Bacterial expression and subsequent purification of epitope scaffold ES5 was performed as previously described [27]. Briefly, purification from inclusion bodies was undertaken under denaturing conditions of 8 M urea, 20 mM Tris-Cl, pH 8.0, 1 mM beta-mercaptoethanol or DTT, followed by nickel chelating chromatography in binding buffer comprised of 8 M urea, 500 mM NaCl, Tris-Cl, pH 8.0, imidazole 10 mM. Washes and elutions were undertaken in binding buffer supplemented with 30 mM and 500 mM imidazole, respectively. Refolding was performed at 4°C by dilution (1∶100) into refolding buffer comprised of 50 mM Tris-Cl, pH 8.0, 250 mM NaCl, 500 mM L-Arginine, 0.1 mM glutathione reduced, 0.01 mM glutathione oxidized, 0.03% N-laurylsarcosine and 0.1 mM ZnCl_2_.

### Construction of adjuvant-containing MPER liposomes

The procedure used to prepare MPER peptide liposomes earlier [19] was employed to construct adjuvant containing MPER liposomes. Briefly, chloroform stocks of POPC, POPE, DMPA and CH were mixed in glass tubes at a molar ratio of 45∶25∶20∶1.33 respectively. The MPER peptide stock (made in chloroform-methanol 7∶3 v/v) solution was added to this mixture to give a final peptide to total lipid ratio of 1∶420. Appropriate volumes of stock solutions of adjuvants MPL-A (in chloroform) and R848 (in methanol) needed to yield desired dose of adjuvants were added to the above lipid mixture. The lipids-peptide-adjuvant mixture was dried under the stream of nitrogen to remove all visible traces of chloroform and methanol. Any residual amount of chloroform and methanol was removed by placing the dried film under vacuum for overnight. The dried film was hydrated by adding appropriate volume of PBS buffer (pH 7.4) and incubating at 37°C for 45 minutes. The hydrated mixture was then sonicated in a bath sonicator (Misonix 3000) and extruded through 0.4 µm and 0.2 µm polycarbonate membranes using either a mini-extruder obtained from Avanti Polar Lipids or a Lipex model thermo barrel (Northern Lipids) extruder that uses high pressure nitrogen gas for extrusion. The extruded liposomes were quality controlled by checking for the binding of 2F5 or both 2F5 and 4E10 depending on the sequence of the MPER peptide as described earlier [19]. The functionally active nature of liposome incorporated MPL-A and R848 was confirmed by the ability of MPER-adjuvant liposomal constructs to induce cytokine release in human PBMC.

### Immunizations

Guinea pigs were purchased from Charles River and housed in the Vivarium at the Duke Human Vaccine Institute Animal Research Facility. Some initial studies in guinea pigs (Figure 1B, 2B and 2E) involved immunization through intramuscular, intranasal and sublingual routes with total dose of 100 µg of immunogen distributed in a 16∶8∶1 ratio respectively. In all other studies, guinea pigs (4 animals per immunogen group) were immunized every three weeks intramuscularly. At each immunization, the JRFL gp140CF group of guinea pigs were administered intramuscularly with 400 µl (in two sites 200 µl/site) of recombinant JRFL gp140CF formulated in 15% emulsigen and oCpG to give a dose of 100 µg per animal of recombinant JRFL gp140 and 50 µg per animal of oCpG. The MPER liposome group of guinea pigs received MPER/MPL-A liposomes formulated in 15% emulsigen and oCpG. The MPER/MPL-A liposome immunization involved intramuscular administration of guinea pigs at two sites (200 µl per site). Each animal received a dose of 100 µg MPER peptide, 200 µg of MPL-A and 50 µg of oCpG respectively. Serum samples were collected 10 days after each immunization and stored at −80°C until use.

Two groups of rhesus macaques were immunized intramuscularly; one with JRFL gp140CF (100 µg per animal) adjuvanted with AS01_B_ (GSK Bio) and the second with MPER liposomes (1 mg MPER peptide per animal) formulated in emulsigen and oCPG (1 mg per animal) at weeks 0, 4, 8, 12 and 16. The ASO1_B_ adjuvant is a GSK-liposome adjuvant system containing MPL-A and QS21. Serum samples were collected at weeks 2, 6, 10, 14 and 18. The JRFL gp140CF immunized rhesus macaques were boosted with MPER/MPL-A/R848 liposomes (1 mg each of MPER peptide, MPL-A and R848 per animal) at weeks 48, 51 and 55. Serum samples were collected at weeks 48, 49, 50, 51, 55 and 56 and were stored at −80°C until use.

### Surface Plasmon Resonance assays for screening of serum samples for MPER specific binding and epitope mapping

The MPER specific binding responses present in immunized animal serum samples were measured using a BIAcore 3000 instrument and data analyses were performed with BIAevaluation 4.1 software (Biacore). Biotinylated 2F5 nominal epitope peptide (Biotin-SP62, QQEKNEQELLELDKWASLWN), 4E10 nominal epitope peptide (Biotin-4E10p, SLWNWFNITNWLWYIK) and a bi-epitope MPER peptide (Biotin-MPER656, NEQELLELDKWASLWNWFNITNWLW) were immobilized (150 RU) on difference flow channels of a Streptavidin chip (Biacore). The activity of immobilized MPER peptides was confirmed by checking the binding of 2F5 or 4E10 mAbs at 5 or 10 µg/ml concentration. The serum samples after 50 fold dilution in PBS buffer (pH 7.4) were injected over the MPER peptide surfaces for 5 minutes at a 30 µl/minute flow rate. The responses were continued to be monitored for 5 minutes after the injection of diluted serum samples. The peptide surfaces were regenerated by flowing over Glycine-HCl pH 2.0. A biotinylated scrambled MPER peptide (NKEQDQAEESLQLWEKLNWL) immobilized on the remaining flow channel served as a negative control surface to subtract out the non-specific interactions of serum sample with the chip. The dissociation phase of the MPER peptide specific binding of sera was biphasic and could be resolved into a faster and slower component. The dissociation rates shown in the figures were derived from the initial faster phase. The gp41 inter proteins (92UG gp41-inter and GCN4-gp41 inter) specific binding responses in rhesus macaques sera were measured by flowing 50 fold diluted rhesus macaques sera over a CM5 chip immobilized with ∼500 RU of recombinant HIV-1 gp41 MN, gp41-inter proteins and HIV-1 p66 protein surfaces. The binding responses on HIV-1 p66 protein surface were used to subtract out responses due to non-specific interactions. The IgGs purified from rhesus macaques sera using Protein G spin columns and desalting columns (Thermo Scientific) following manufacturer's recommended protocol were flowed at a 100 µg/ml concentration over the gp41-inter proteins surfaces. The rhesus macaque serum IgGs (150 µg/ml) binding to 2F5 epitope scaffolds was performed using a CM5 chip immobilized with ∼1500 RU of ES proteins.

Epitope mapping of MPER specific responses found in immunized animal sera was carried out using a BIAcore A100 instrument and Streptavidin chip (Biacore). The biotinylated 2F5 nominal epitope peptide (Biotin-SP62) and seven of the biotinylated alanine scanning mutant MPER peptides were immobilized in duplicate on 16 different spots on four flow channels of the chip. The median spot on each flow channel served as a blank surface to subtract out non-specific interactions. The 50 fold diluted serum samples were injected over the native and mutant MPER peptide and reference surfaces for 5 minutes at a 30 µl/minute flow rate. The dissociation phase was monitored for 10 minutes and the surfaces were regenerated using Glycine-HCl pH 2.0 between cycles. The responses at the end of the dissociation phase (450–500 seconds after the injection of serum samples was over) were averaged and used to calculate normalized binding response which is defined as the ratio between the average binding response to a mutant peptide and the corresponding binding response to the native peptide.

Cardiolipin binding of 2F5, 13H11 mAbs and purified IgGs from rhesus macaques sera were performed using POPC-Cardiolipin (25∶75) liposomes and Biacore L1 chip as described earlier [53].

### ELISA assays for MPER binding

Direct binding ELISAs were conducted in 96 well ELISA plates coated with 0.2 µg/well antigen (WT 2F5 epitope peptide SP62) in 0.1 M sodium bicarbonate and blocked with assay diluent (PBS containing 4% (w/v) whey protein/15% Normal Goat Serum/0.5% Tween20/0.05% Sodium Azide). Sera were incubated for 1 hour in two fold serial dilutions beginning at 1∶25 followed by washing with PBS/0.1% Tween-20. 100 µl Alkaline phosphatase conjugated goat anti-human secondary antibody was incubated for 1 hour, washed and detected with 100 µl substrate (Carbonate-BiCarbonate (CBC) buffer+2 mM MgCl_2_+1 mg/ml p-npp [4-Nitrophenyl phosphate di(2-amino-2-ethyl-1,3-propanediol) salt]). Plates were read at 405 nm, 45 minutes.

### Neutralization assay

The neutralizing activity of immune sera was determined by monitoring reductions in luciferase (Luc) reporter-gene expression after a single round of infection by pseudotyped HIV-1 viruses in TZM-bl cells, as described previously [52].

## Supporting Information

Figure S1 **JRFL gp140CF specific responses mounted by rhesus macaques immunized with JRFL gp140CF.** ELISA endpoint titer of immunized rhesus macaques sera showing binding to JRFL gp140CF protein at different time points.
(TIF)

Figure S2 **JRFL gp140 immunized rhesus macaques mount gp41 specific responses that target a post-fusion conformation of gp41.** **A–D:** SPR sensogram of rhesus macaques serum IgG (100 µg/ml) binding to recombinant gp41 MN (broken lines), gp41-inter (solid lines) and GCN4-gp41 inter (dotted lines) and (broken lines). The IgGs purified from Week 14 sera of rhesus macaques were used.
(TIF)

Figure S3 **11F10 mAb interaction with gp41-inter and recombinant gp41 proteins.** SPR sensogram of binding of 11F10 mAb at 2 (red), 5 (blue), 10 (green) and 20 (gray) µg/ml concentration to (**A**) GCN4-gp41 inter, (**B**) 92UG gp41-inter and (**C**) recombinant gp41 MN immobilized on a Biacore CM5 chip is shown. The binding curves were fitted globally to a 1∶1 Langmuir model to obtain the displayed rate constants and dissociation constant. The best fit are overlaid in black.
(TIF)

Figure S4 **Binding of 11F10 mAb to anionic phospholipids.** A comparison of 11F10 (red), 2F5 (green) and 13H11 (blue) mAbs binding responses to (**A**) phospatidylserine and (**B**) cardiolipin containing liposomes is shown. The mAbs at 100 µg/ml concentration were flowed over POPC-POPS (25∶75) and POPC-Cardioipin (25∶75) liposomes captured on a Biacore L1 chip.
(TIF)

Figure S5 **Phospholipid binding of rhesus macaques serum IgG.** **A:** SPR sensogram of rhesus macaque (# 41546) serum IgG (100 µg/ml) from week 0 (pre-bleed), 48 (pre-liposome boost bleed) and 52 (post-liposome boost bleed) binding to cardiolipin containing liposomes (POPC:cardiolipin 25∶75) are shown. The binding responses of 2F5 and 13H11 mAbs are shown as comparison. **B:** The cardiolipin binding responses obtained for all rhesus macaques serum IgG pre and post vaccination are shown and compared with the binding responses of 2F5 and 13H11 mAbs. The binding responses of post vaccination IgG (weeks 48–56) shown are week 0 bleeds response subtracted.
(TIF)

Figure S6 **Schematics of protein and peptide constructs.** **A:** The JRFL gp140CF construct had the cleavage site, fusion domain, transmembrane (TM) and cytoplasmic domains deleted from the full length precursor gp160 sequence. The three residues at the C-terminal are indicated. The construct was made using methods reported earlier (Liao et al 2006). Blue-native PAGE displayed on the right indicates the oligomeric nature of JRFL gp140CF. **B:** A pictorial representation of MPER peptide-liposome is shown. The liposomes were made using protocols described earlier (Dennison et al 2009). **C:** The MPER sequence of JRFL gp140CF is aligned with the sequence of MPER peptide in the MPER656 liposome construct. The differences are coded in red.
(TIF)

Figure S7 **Schematic of gp41 fusion intermediate protein constructs.** The design and trimer organization of gp41 inter proteins constructs used here to test the gp41 conformations targeted by rhesus macaque IgGs is shown for (A) gp41- inter that contains a six-helix bundle at the N-terminal and (B) GCN4-gp41 inter that has a trimeric GCN4 coiled coil and were described in detail earlier (Frey et al 2008 and Frey et al 2010). HR2, heptad repeat 2; HR1, heptad repeat 1; C-C loop, immunodominant region with a disulfide bond; MPER, membrane proximal exernal region; fd, trimerization fold on tag.
(TIF)

Figure S8 **MPER peptide-liposome binding of mAbs and rhesus macaques serum IgG.** **A:** Mutation of hydrophobic residues in CDR H3 loop of recombinant 2F5 mAb (L100aA, F100bA and L100aA/F100bA) impedes binding to MPER peptide-liposomes. The R95A mutation at the base of the CDR H3 loop that was designed and demonstrated to disrupt gp41 binding (Alam et al 2009) showed no binding to MPER peptide-liposomes. **B:** A comparison of the binding of MPER mabs 2F5, 11F10 and 13H11 to MPER peptide-liposomes is shown. **C–F:** Rhesus macaques serum IgG of animals 03525 (C), 41546 (D), 51834 (E) and 51902 (F) to MPER peptide-liposomes are shown for pre-immune (week 0) and post-MPER656 liposomes boost bleeds. The sensograms shown were obtained by flowing mAbs and serum IgGs at a 100 µg/ml concentration over MPER liposomes (∼500 RU) captured on a Biacore L1 chip. The non-specific binding responses to the peptide-free liposomes captured on a parallel flow cell in the same L1 chip were subtracted to obtain specific binding responses shown in the panels A through F.
(TIF)

Figure S9 **The gp41 MPER specific antibody responses in guinea pigs following double prime-boost immunization.** **A:** Scheme showing the prime-boost immunization strategy. For the sake of clarity the time-line of immunization and blood-draw is not shown. MPER liposomes containing 2F5 nominal epitope peptide were used in immunizations 1 through 4 and 6 through 8. For immunizations 10 and 11 a longer MPER peptide (MPER656) liposomes containing both 2F5 and 4E10 epitopes were the preferred immunogen. Both MPER liposomes contained MPL-A adjuvant embedded in them. **B:** ELISA endpoint titer of the 2F5 epitope peptide (SP62 peptide) specific responses in guinea pigs sera from different bleeds. **C:** The 2F5 epitope peptide (SP62 peptide) specific responses of guinea pigs sera determined by SPR are shown as a function of post-bleed number. P1-P11, post-immune bleeds 1-11. **D:** SPR sensogram displaying the comparison of 2F5 epitope peptide (SP62 peptide) specific binding of guinea pig 1375 sera from different bleeds with 10µg/ml 2F5 mAb is shown as representative data. **E:** Epitope mapping of post-immune bleed 6-11 of guinea pig 1375 is shown in comparison to 2F5 mAb. The normalized binding shown is the ratio between binding responses of sera to the alanine scanning mutant peptides and WT 2F5 epitope peptide SP62. The dotted circle highlights the mapping of MPER specific responses in guinea pig 1375 sera to D^664^KW^666^ residues as did 2F5 mAb. Data is representative of at least two measurements on adjacent spots on the same sensor chip.
(TIF)
