# Supplementary figures and images for: Induction of Antibodies in Rhesus Macaques That Recognize a Fusion-Intermediate Conformation of HIV-1 gp41
Source: PLoS One. 2011 Nov 30;6(11):e27824. doi: 10.1371/journal.pone.0027824 (PMC3227606; doi:10.1371/journal.pone.0027824)

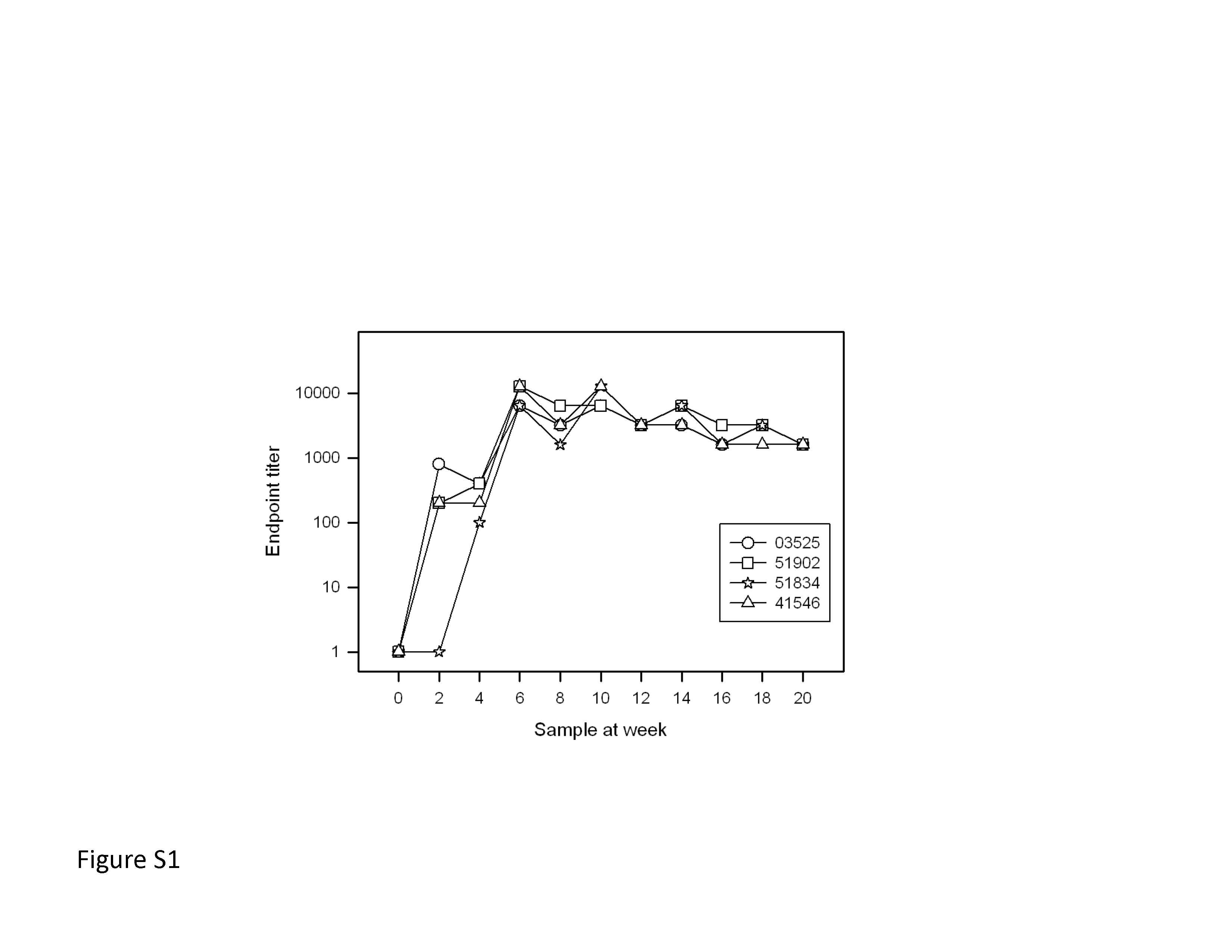

Supplement: Figure S1 — JRFL gp140CF specific responses mounted by rhesus macaques immunized with JRFL gp140CF. ELISA endpoint titer of immunized rhesus macaques sera showing binding to JRFL gp140CF protein at different time points. (TIF) [file pone.0027824.s001.tif]

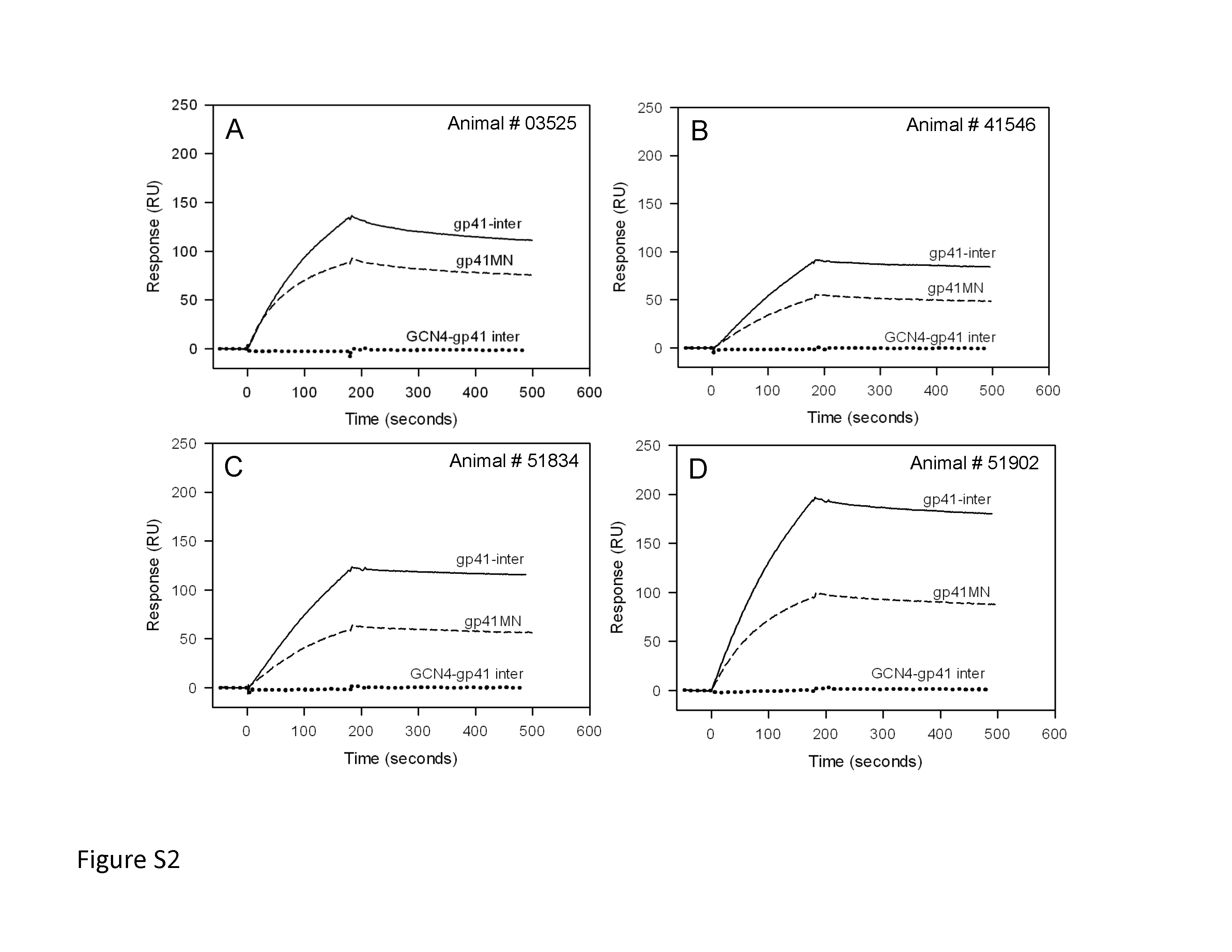

Supplement: Figure S2 — JRFL gp140 immunized rhesus macaques mount gp41 specific responses that target a post-fusion conformation of gp41. A–D: SPR sensogram of rhesus macaques serum IgG (100 µg/ml) binding to recombinant gp41 MN (broken lines), gp41-inter (solid lines) and GCN4-gp41 inter (dotted lines) and (broken lines). The IgGs purified from Week 14 sera of rhesus macaques were used. (TIF) [file pone.0027824.s002.tif]

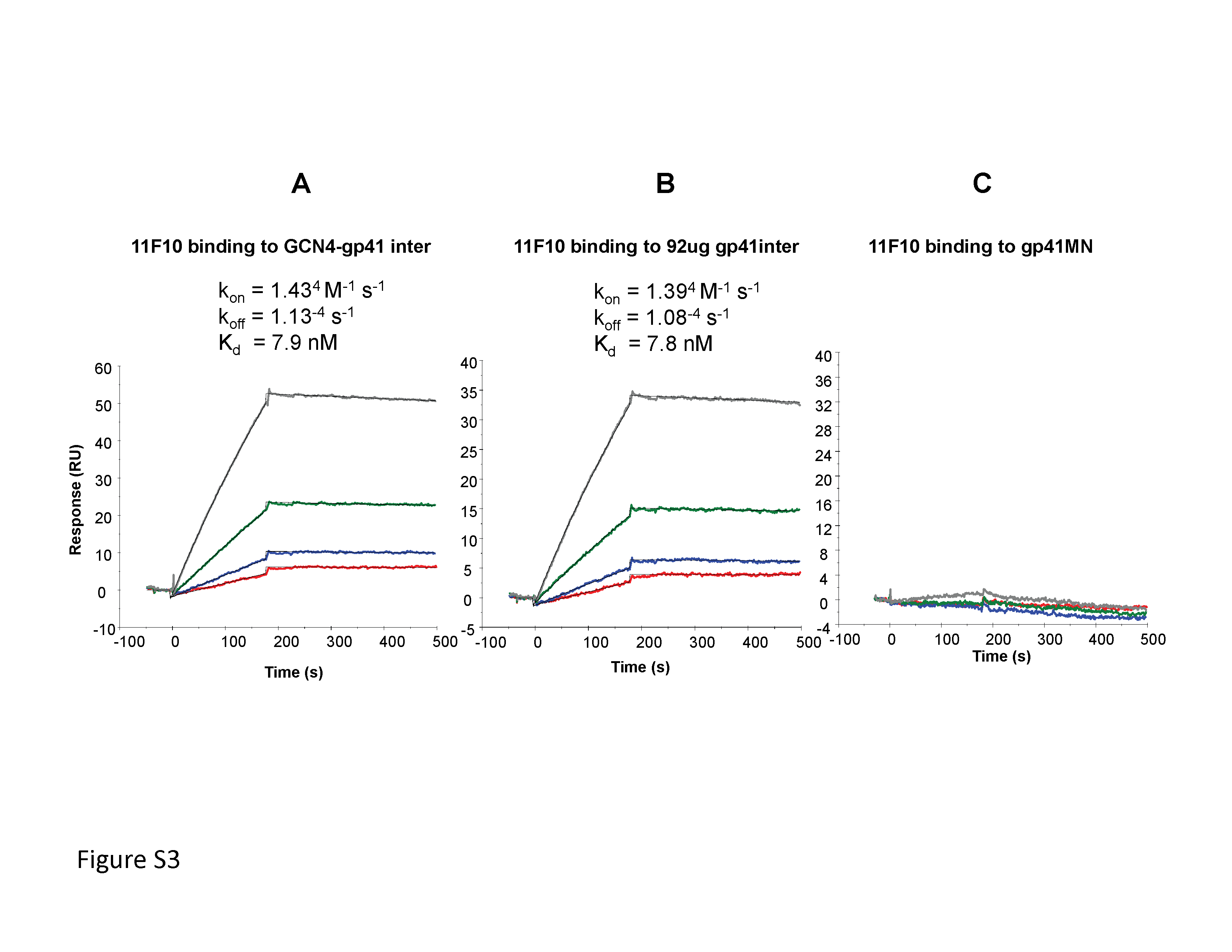

Supplement: Figure S3 — 11F10 mAb interaction with gp41-inter and recombinant gp41 proteins. SPR sensogram of binding of 11F10 mAb at 2 (red), 5 (blue), 10 (green) and 20 (gray) µg/ml concentration to (A) GCN4-gp41 inter, (B) 92UG gp41-inter and (C) recombinant gp41 MN immobilized on a Biacore CM5 chip is shown. The binding curves were fitted globally to a 1∶1 Langmuir model to obtain the displayed rate constants and dissociation constant. The best fit are overlaid in black. (TIF) [file pone.0027824.s003.tif]

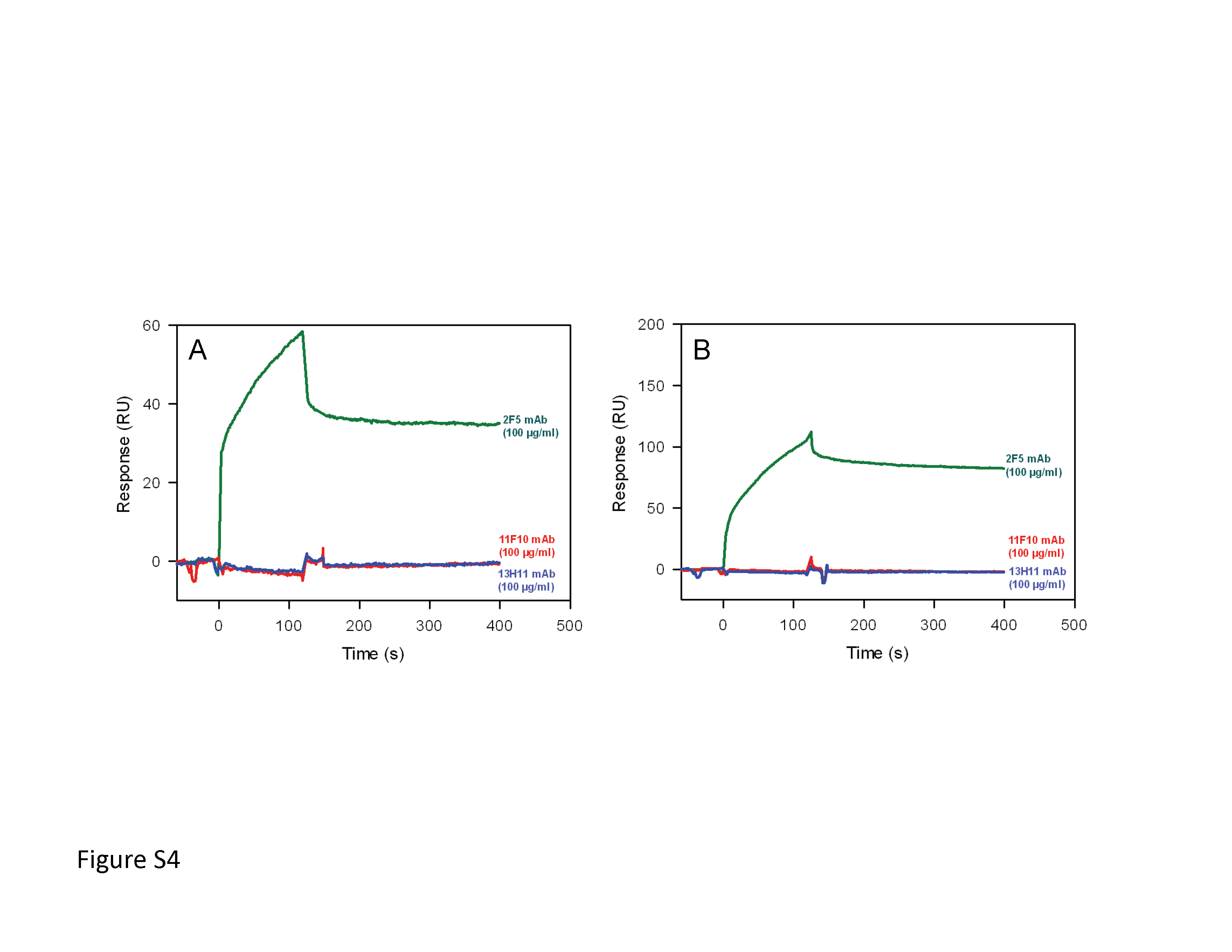

Supplement: Figure S4 — Binding of 11F10 mAb to anionic phospholipids. A comparison of 11F10 (red), 2F5 (green) and 13H11 (blue) mAbs binding responses to (A) phospatidylserine and (B) cardiolipin containing liposomes is shown. The mAbs at 100 µg/ml concentration were flowed over POPC-POPS (25∶75) and POPC-Cardioipin (25∶75) liposomes captured on a Biacore L1 chip. (TIF) [file pone.0027824.s004.tif]

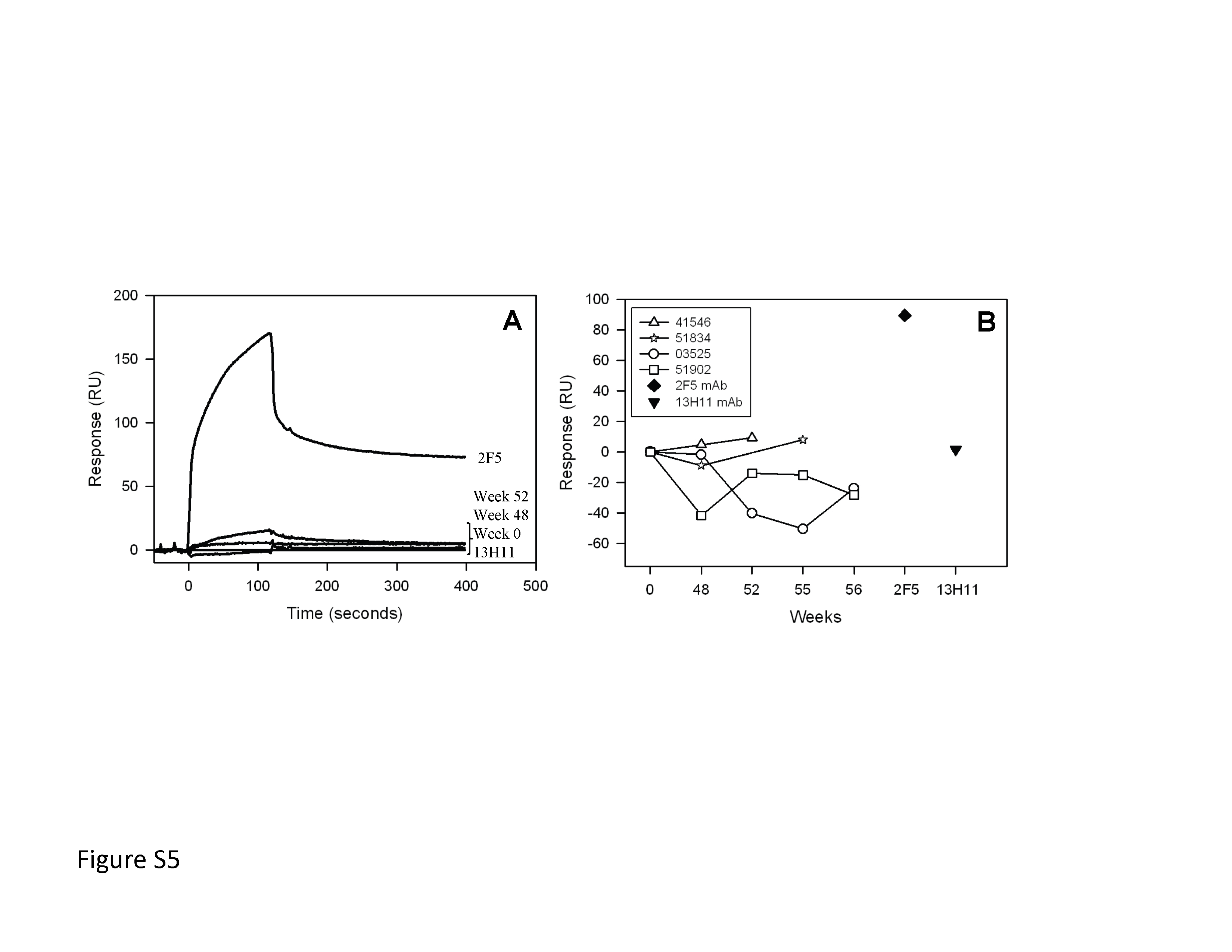

Supplement: Figure S5 — Phospholipid binding of rhesus macaques serum IgG. A: SPR sensogram of rhesus macaque (# 41546) serum IgG (100 µg/ml) from week 0 (pre-bleed), 48 (pre-liposome boost bleed) and 52 (post-liposome boost bleed) binding to cardiolipin containing liposomes (POPC:cardiolipin 25∶75) are shown. The binding responses of 2F5 and 13H11 mAbs are shown as comparison. B: The cardiolipin binding responses obtained for all rhesus macaques serum IgG pre and post vaccination are shown and compared with the binding responses of 2F5 and 13H11 mAbs. The binding responses of post vaccination IgG (weeks 48–56) shown are week 0 bleeds response subtracted. (TIF) [file pone.0027824.s005.tif]

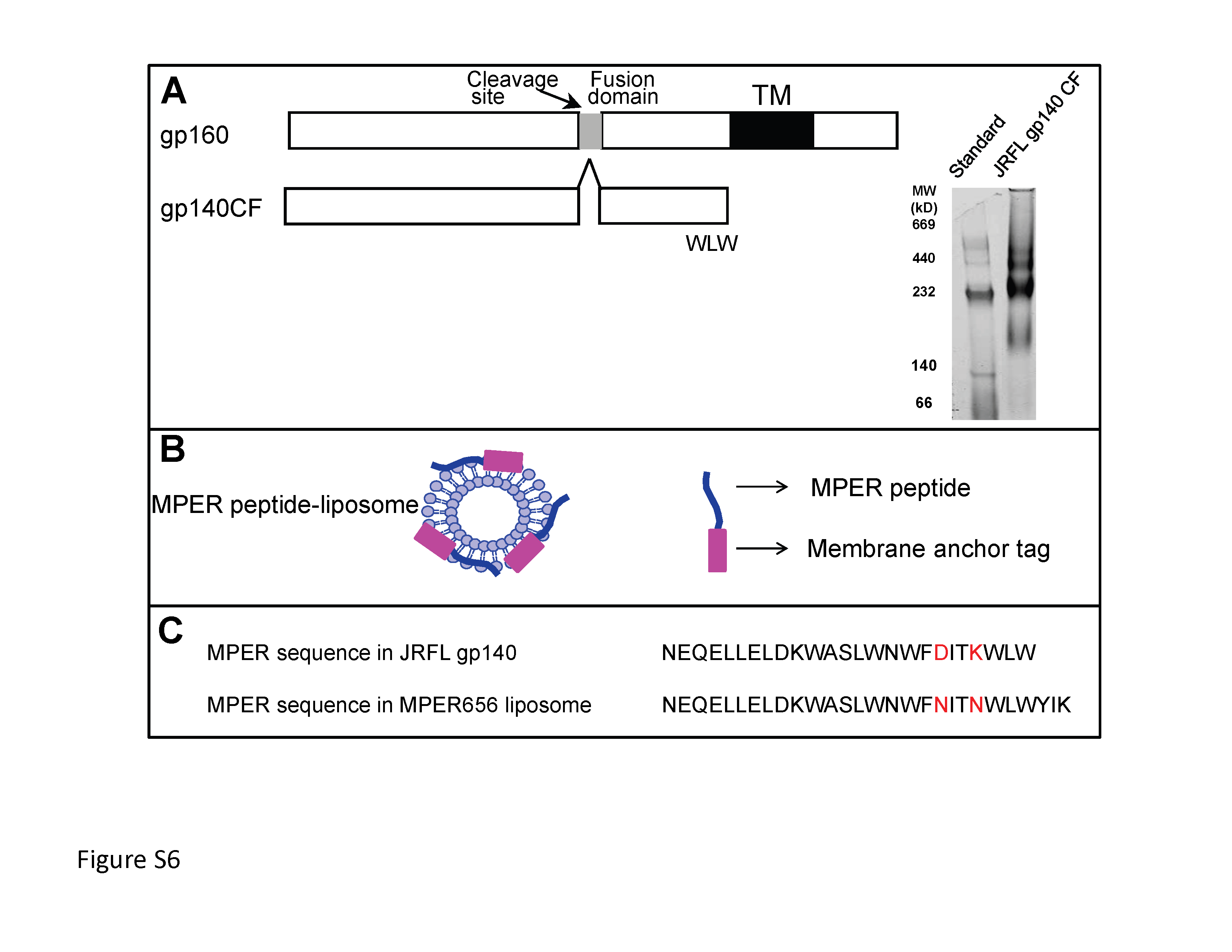

Supplement: Figure S6 — Schematics of protein and peptide constructs. A: The JRFL gp140CF construct had the cleavage site, fusion domain, transmembrane (TM) and cytoplasmic domains deleted from the full length precursor gp160 sequence. The three residues at the C-terminal are indicated. The construct was made using methods reported earlier (Liao et al 2006). Blue-native PAGE displayed on the right indicates the oligomeric nature of JRFL gp140CF. B: A pictorial representation of MPER peptide-liposome is shown. The liposomes were made using protocols described earlier (Dennison et al 2009). C: The MPER sequence of JRFL gp140CF is aligned with the sequence of MPER peptide in the MPER656 liposome construct. The differences are coded in red. (TIF) [file pone.0027824.s006.tif]

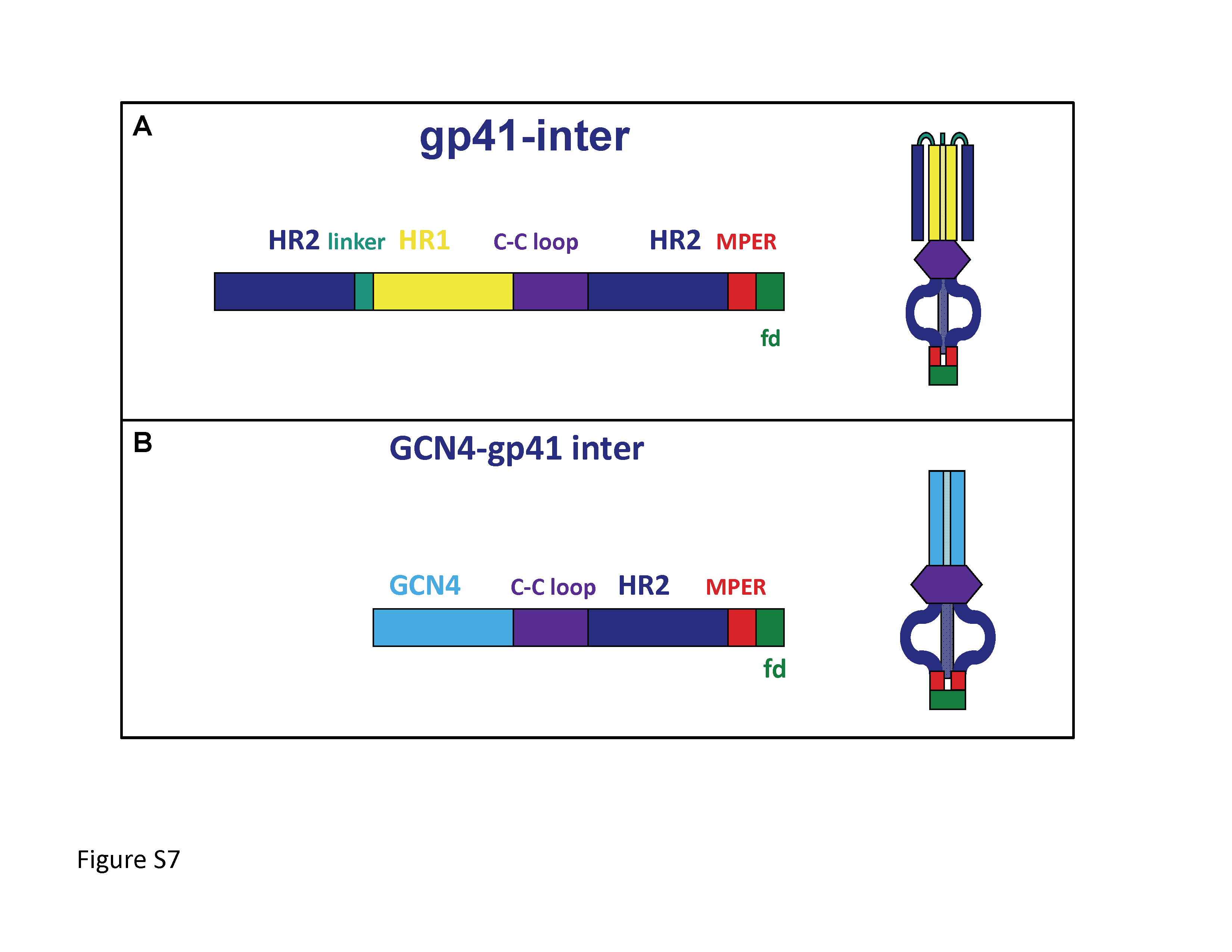

Supplement: Figure S7 — Schematic of gp41 fusion intermediate protein constructs. The design and trimer organization of gp41 inter proteins constructs used here to test the gp41 conformations targeted by rhesus macaque IgGs is shown for (A) gp41- inter that contains a six-helix bundle at the N-terminal and (B) GCN4-gp41 inter that has a trimeric GCN4 coiled coil and were described in detail earlier (Frey et al 2008 and Frey et al 2010). HR2, heptad repeat 2; HR1, heptad repeat 1; C-C loop, immunodominant region with a disulfide bond; MPER, membrane proximal exernal region; fd, trimerization fold on tag. (TIF) [file pone.0027824.s007.tif]

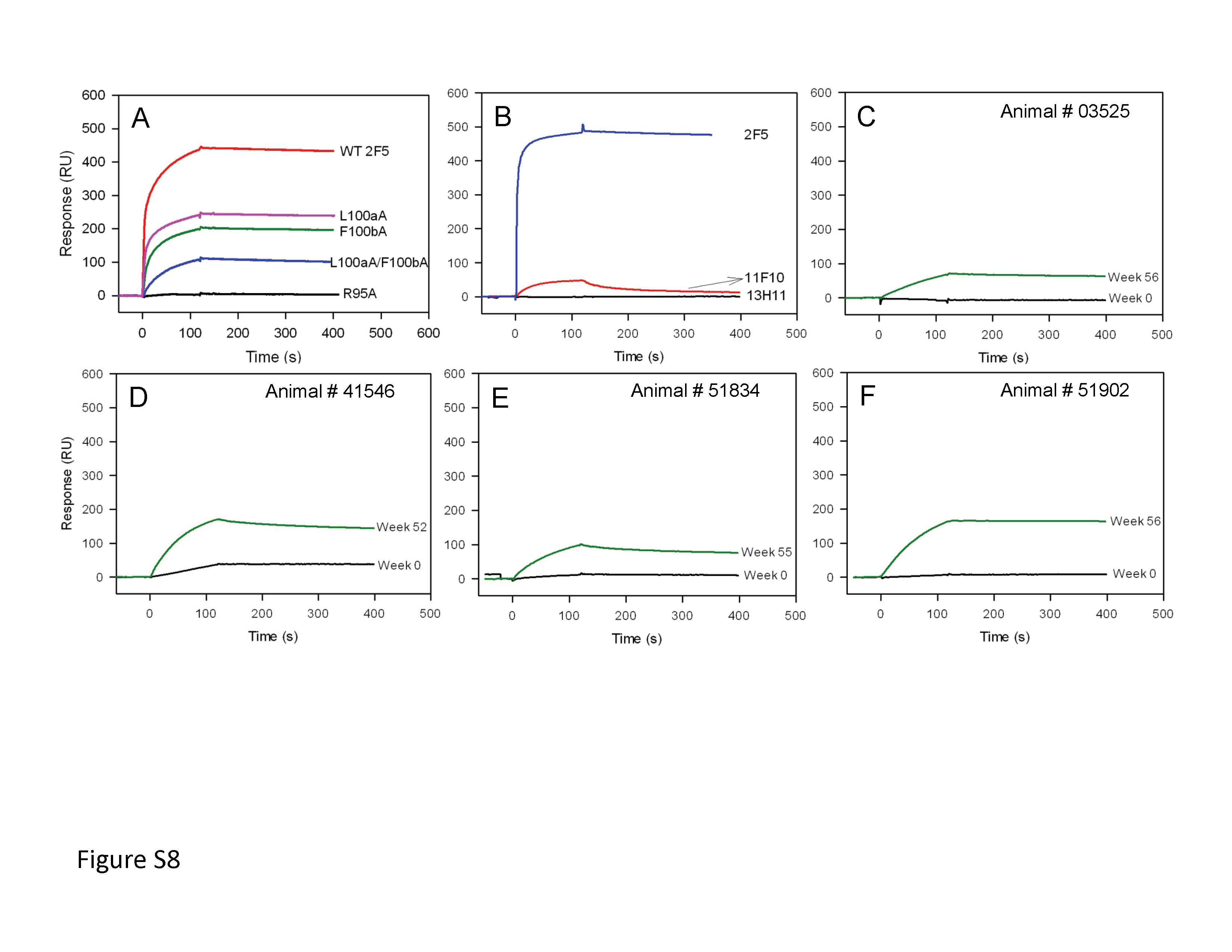

Supplement: Figure S8 — MPER peptide-liposome binding of mAbs and rhesus macaques serum IgG. A: Mutation of hydrophobic residues in CDR H3 loop of recombinant 2F5 mAb (L100aA, F100bA and L100aA/F100bA) impedes binding to MPER peptide-liposomes. The R95A mutation at the base of the CDR H3 loop that was designed and demonstrated to disrupt gp41 binding (Alam et al 2009) showed no binding to MPER peptide-liposomes. B: A comparison of the binding of MPER mabs 2F5, 11F10 and 13H11 to MPER peptide-liposomes is shown. C–F: Rhesus macaques serum IgG of animals 03525 (C), 41546 (D), 51834 (E) and 51902 (F) to MPER peptide-liposomes are shown for pre-immune (week 0) and post-MPER656 liposomes boost bleeds. The sensograms shown were obtained by flowing mAbs and serum IgGs at a 100 µg/ml concentration over MPER liposomes (∼500 RU) captured on a Biacore L1 chip. The non-specific binding responses to the peptide-free liposomes captured on a parallel flow cell in the same L1 chip were subtracted to obtain specific binding responses shown in the panels A through F. (TIF) [file pone.0027824.s008.tif]

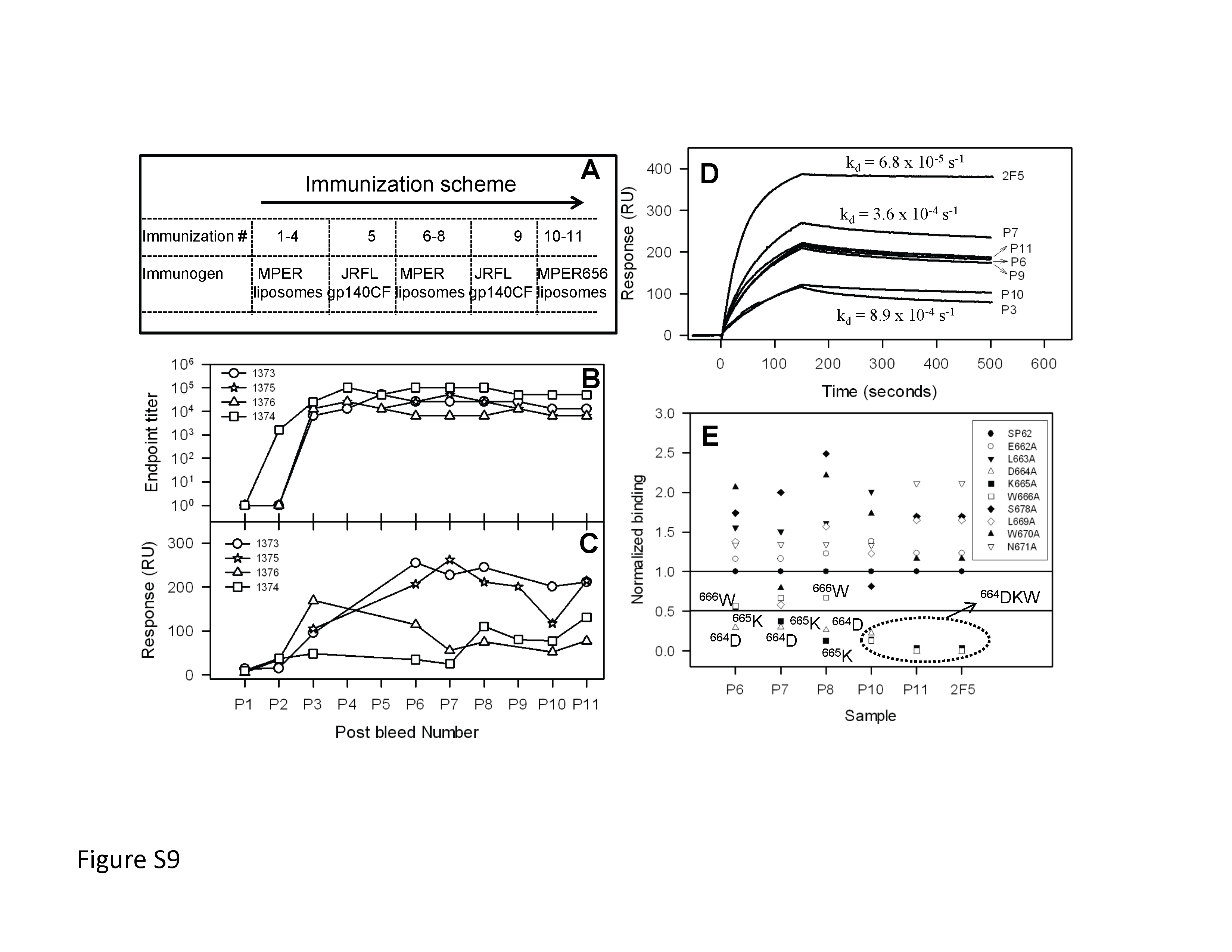

Supplement: Figure S9 — The gp41 MPER specific antibody responses in guinea pigs following double prime-boost immunization. A: Scheme showing the prime-boost immunization strategy. For the sake of clarity the time-line of immunization and blood-draw is not shown. MPER liposomes containing 2F5 nominal epitope peptide were used in immunizations 1 through 4 and 6 through 8. For immunizations 10 and 11 a longer MPER peptide (MPER656) liposomes containing both 2F5 and 4E10 epitopes were the preferred immunogen. Both MPER liposomes contained MPL-A adjuvant embedded in them. B: ELISA endpoint titer of the 2F5 epitope peptide (SP62 peptide) specific responses in guinea pigs sera from different bleeds. C: The 2F5 epitope peptide (SP62 peptide) specific responses of guinea pigs sera determined by SPR are shown as a function of post-bleed number. P1-P11, post-immune bleeds 1-11. D: SPR sensogram displaying the comparison of 2F5 epitope peptide (SP62 peptide) specific binding of guinea pig 1375 sera from different bleeds with 10µg/ml 2F5 mAb is shown as representative data. E: Epitope mapping of post-immune bleed 6-11 of guinea pig 1375 is shown in comparison to 2F5 mAb. The normalized binding shown is the ratio between binding responses of sera to the alanine scanning mutant peptides and WT 2F5 epitope peptide SP62. The dotted circle highlights the mapping of MPER specific responses in guinea pig 1375 sera to D664KW666 residues as did 2F5 mAb. Data is representative of at least two measurements on adjacent spots on the same sensor chip. (TIF) [file pone.0027824.s009.tif]
